# How to deal with the consent of adults with cognitive impairment involved in European geriatric living labs?

**DOI:** 10.1186/s13010-021-00101-1

**Published:** 2021-06-16

**Authors:** Guillaume Sacco, Frédéric Noublanche, Frédéric Blazek, Catherine Hue, Loïc Carballido, Marine Asfar, Philippe Allain, Cédric Annweiler

**Affiliations:** 1 Department of Geriatric Medicine and Memory Clinic, Research Center on Autonomy and Longevity, University Hospital, Angers, France; 2 Univ Angers, Université de Nantes, LPPL, SFR CONFLUENCES, F-49000, Angers, France; 3 Université Côte d’Azur, CoBTek, Nice, France; 4 Direction, University Hospital, Angers, France; 5 Robarts Research Institute, Department of Medical Biophysics, Schulich School of Medicine and Dentistry, The University of Western Ontario, London, ON Canada

**Keywords:** Living lab, Geriatrics, Older adults, Consent, Ethics

## Abstract

**Background:**

Living labs are realistic environments designed to create links between technology developers and end-users (i.e. mostly older adults). Research in LLH (Living labs in health) covers a wide range of studies from non-interventional studies to CT (clinical trials) and should involve patients with neurocognitive disorders. However, the ethical issues raised by the design, development, and implementation of research and development projects in LLH have been the subject of only little interest thus far.

**Objective:**

Our aim was to determine a pragmatic, ethical and regulatory correct approach to seek the informed consent of patients with neurocognitive disorders according to the different types of studies carried out in European LLH, with a focus on the French context.

**Methods:**

A narrative review of regulatory texts and clinical articles was conducted, and a pragmatic procedure to determine the decision-making capacity of older adults in LLH was proposed.

**Results:**

Individuals must be adequately informed and freely agree to participate in CT. The capacity to consent should be assessed in CT including cognitively impaired older adults. We propose the following steps: first to assess for delirium using the 4 ‘A’s Test (4AT) or the 3-min Diagnostic interview for Confusion Assessment Method (3D-CAM), second to search for medical history of major neurocognitive disorder, and third to assess the decision capacity using the University of California, San Diego Brief Assessment of Capacity to Consent (UBACC).

**Conclusions:**

Including individuals with neurocognitive disorders in research implies using an efficient and pragmatic strategy to inform participants and obtain their consent. The tool we offer here may be useful in the routine operation of LLH but can also be extended to all CT with this population.

## Introduction

The increases in life expectancy and in the proportion of very old, polymorbid, frail and disabled adults generate new needs. Today it proves urgent to radically innovate and offer new services or divert new uses to meet these needs. The challenge is to innovate efficiently, and in line with the real needs of users, in their diversity. Living labs in health (LLH) are precisely thought to fill the gap between the solutions proposed by developers of new uses or technologies and the real needs of users with specific health issues. Living labs are realistic environments designed to create links between technology developers and end-users. Living labs enable end-users to communicate their needs and expectations directly to technology developers (co-design), and to participate in prototype testing (codevelopment) [1, 2]. LLH appear particularly appropriate to vulnerable groups, including older adults with diseases and/or loss of function [3].

Research in LLH covers a wide range of studies from non-interventional studies to clinical trials. However, the ethical issues raised by the design, development, and implementation of research and development projects in LLH have been the subject of only little interest thus far [4, 5]. This question is not, however, so simple. For instance, in our ALLEGRO LLH, in which real sick older patients are hospitalized to allow them participating to tests in real conditions [6], a number of patients with neurocognitive disorders are still excluded from the LLH process because of the lack of standardized procedure to determine their incapacity to consent. This is all the less acceptable as these exclusions go against the objective of ALLEGRO, which was precisely to include in the process of innovation all real-life older patients. Therefore, older adults with neurocognitive disorders should be integrated in research projects, and are able to participate [7]. This is also in line with guidance related to work with older and/or disabled adults [8–16]. In this context, particular attention should therefore be paid to the ethical issues raised by cognitively-impaired older adults [5, 7, 17]. Questioning the need for including such people in studies should be thought during the design of the study and validating by ethics committees. Our aim here was to determine a pragmatic, ethical and regulatory correct approach to seek the informed consent of patients with neurocognitive disorders according to the different types of studies carried out in LLH.

## Regulatory reminders for non-interventional research

In France, non-interventional research must be divided in two categories. On the one hand, research implying human being (i.e. category 3 of the article L1121–1 from the French public health code), on the other hand research on data.

Research on data is not considered as a research implying human being. It concerns only observational researches on health data collected in usual heath care and researches without collection of health data (e.g. questionnaires with healthcare professionals or satisfaction surveys). Regulation concerning research on data is provided by the regulation (EU) 679/2016 on general data protection (GDPR) [18]. This regulation explains that personal data shall be processed lawfully i.e., the individual is expected to give consent to the processing of personal data for one or more specific purposes (article 6), fairly, and in a transparent manner in relation to the data subject (article 5). Moreover, it is necessary to assess whether the consent is freely given (article 7). Thus, even if not clearly defined as “informed”, the capacity to consent remains at the center of the decisional process. Nevertheless, for data concerning research (Article 21.6 of the GDPR) the person “shall have the right to object to processing of personal data concerning him or her”. Moreover, article 9.4 state that “member States may maintain or introduce further conditions, including limitations, with regard to the processing of genetic data, biometric data or data concerning health”. In France, according to the article 73 from the law n°78–17 of January 6th. 1978 (law informatics and freedoms), patients could have only general information regarding the research project and not objecting to processing their personal data.

The Oviedo convention [19, 20] frame researches implying human being in non-interventional studies as this convention is applying for all researches in biology and heath. It concerns studies without specific risks, constraints or modification of usual care for the patient (i.e. observational prospective studies). For researches implying human being in non-interventional studies, the patient should be informed regarding the research. Expression of consent differ across European countries from the non-express opposition in France (article L1122.1.1 from the French public health code), to informed consent (e.g. in Belgium).

## Regulatory reminders for clinical trials

As detailed by Brugère and Gzil [21], several regulatory texts govern the informed consent in clinical trials. “Informed consent” is the voluntary agreement of an individual, or the legally acceptable representative, who has the legal capacity to give consent, and who exercises free power of choice, without undue inducement or any other form of constraint or coercion to participate in research [22]. The individual must have sufficient knowledge and understanding of the nature of the proposed research, the anticipated risks and potential benefits, and the requirements of the research to be able to make an informed decision. The main regulatory texts used in Europe are the declaration of Helsinki from the world medical association [23]; the Oviedo convention [19, 20], the guideline for good clinical practice E6(R2) from the International Council for Harmonization of Technical Requirements for Pharmaceuticals for Human Use [24]; the regulation (EU) 536/2014 on clinical trials on medicinal products for human use [25], and the regulation (EU) 679/2016 on general data protection [18] from the European Parliament. In France, clinical trials are divided in two categories (article L1121–1 French health public code): a) category 1 are interventional researches including intervention on human being which is no related to usual care, b) category 2 are interventional researches with only low risks and constraints.

For clinical trials including cognitively healthy older adults, the guideline is as follows: i) the individual is adequately informed (article L1122–1 French health public code); ii) the individual freely agrees to participate (in the French law, written consent is mandatory for category 1 clinical trials contrarily to category 2 clinical trial, were consent could be unwritten; article L1122-1-1 health public code); iii) the consent is regularly repeated.

For clinical trials including cognitively-impaired older adults (article L1122–2 French health public code), it must first be determined whether the clinical trial relates directly to a medical condition from which the individual suffers, and whether the research cannot instead be conducted with individuals capable of providing informed consent. The investigator must seek i) informed consent of the legally acceptable representative, and ii) the assent of the patient whenever possible [23]. “Assent” is a term used to express willingness to participate in research by persons who are not able to give informed consent but who are able to understand the proposed research in general, its expected risks and possible benefits, and the activities expected of them as participants. Assent by itself is not enough and must be supplemented with informed consent of the legally acceptable representative. To be as close as possible to the will of the individual losing autonomy, this substituted decision refers to the concept of authenticity [26]. Unlike autonomy, which is ability to provide informed consent, the concept of authenticity is based on the individual’s values (hopes, beliefs, commitments and relationships), and allows substituted judgment, in which the surrogate attempts to decide what the patient ‘would have chosen’. Thus, a decision can be authentic even when made by a surrogate, because authenticity does not require intact capacity for self-determination, only that the decision conforms to the individual’s values [26].

Thus, whether for non-interventional or interventional studies, the whole question is to assess the decision-making capacity of potential participants with neurocognitive disorders in order to determine whether they are able to consent by themselves.

## Proposal of a pragmatic procedure to screen for decision-making incapacity of older adults in LLH

**Fig. 1:**
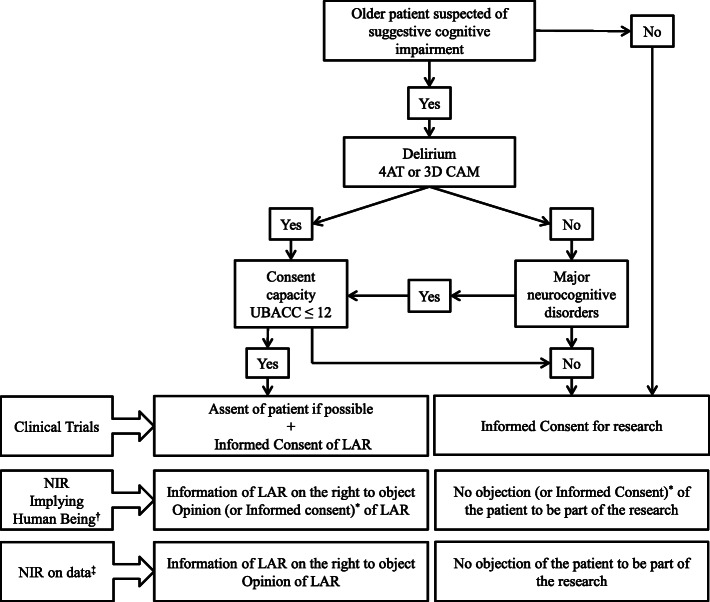
Proposal of a pragmatic approach to determine how to manage the consent for research of older adults with suspected suggestive cognitive impairment. 3D CAM:3 min Diagnostic interview for Confusion Assessment Method; 4AT: the 4 ‘A’s Test; LAR: Legally Acceptable Representative; NIR: Non-Interventional Research; UBACC: the University of California, San Diego Brief Assessment of Capacity to Consent; * depending on each local regulation; † It concerns only observational researches on health data collected in usual heath care and researches without collection of health data (e.g. questionnaires with healthcare professionals or satisfaction surveys); ‡ It concerns studies without specific risks, constraints or modification of usual care for the patient (i.e. observational prospective studies)

The procedure we propose here is based on our experience as clinicians, has been designed to be routinely feasible, and is consistent with regulatory texts (Fig. 1).

First, we propose that individuals should be screened for delirium, as up to 30% of older patients over 80 years suffer from delirium in geriatric acute care units [27], and because more than 60% of delirium are undiagnosed [28] (Fig. 1). The screening for delirium should be both accurate and feasible i.e., with a short administration time and a high negative predictive value. Several tools are proposed [29, 30], including the confusion assessment method (CAM) [31–33], the 3-min Diagnostic interview for Confusion Assessment Method (3D-CAM) [34] or the 4 ‘A’s Test (4AT) [35, 36]. The advantage of the 4AT is that it does not require any training period and its administration takes less than 2 min [35]. Moreover, it exhibits a high negative predictive value, between 96% for the English version [36] and 98% for the French version [37].

The issue most frequently met in ALLEGRO is the presence of major neurocognitive disorders, with at least 40% of patients in geriatric acute care units suffering from major cognitive disorders [38]. Thus, as illustrated in Fig. 1, the second step should be to look for the medical history of major neurocognitive disorders.

Third, the capacity to consent should be assessed according to the medical history of major neurocognitive disorders and to the diagnosis of delirium.

Of note, the decision-making capacity varies among patients with neurocognitive disorders [39], and 30% of cognitively-impaired patients are actually deemed capable to consent [40]. Several tools are proposed to assess the capability to consent [41, 42]. The MacArthur competence assessment tool for clinical research (MacCAT-CR) [43] is the most cited one but it requires 15–20 min of administration, and also a substantial training to certify the administration and interpretation. In contrast, the University of California Brief Assessment of Capacity to Consent (UBACC) [44] requires less than 5 min of administration. A French version has been validated with older adults suffering from neurocognitive disorders; with a sensibility and a specificity of 100% using a cut-off ≤12/20 [45].

Finally, as discussed above, consent should be sought either from the individual when the decision-making capacity is preserved or from the legal representative with the assent of the individual when the decision-making capacity is altered (Fig. 1). If divergent opinions occur between the legally acceptable representatives of a patient considered as having altered decision-making capacity, we are recommending stopping the research process and to continue the standard cares as they are the best-known cares.

## Making information accessible and understandable to individuals with neurocognitive disorders

Educational psychology literature suggests that effective learning can be improved by multimodal presentation [46]. Such multimodal approach has driven Dewing to help patients better understanding studies information [47]. In particular, video has been used to improve informed consent in cognitively healthy patients [48–51]. This media seems to improve understanding also in participants with low education [52] and psychiatric disorders [53]. However, the most recent systematic review reports that the benefit of audio-visual support remains unclear to enhance informed consent [54]. In any case, it is imperative to ensure that the individual and relatives have understood the issue, the benefits and the risks of the studies that are offered to them in the LLH. Novel approaches using mHealth and interactive informed consent could be an attractive option in the near future [55].

## Ensuring over time that the participant remains consenting to the study

When undertaking research with individuals with neurocognitive disorders, the literature introduces the concepts of “process consent” [47] or “rolling consent” [56, 57] to ensure willing participation. These include i) the necessity to repeat information on an iterative basis (i.e., not only when requested) and asking for consent (or assent) during the various stages of the research development; ii) listening to the content and nuances of the speech and continuously assessing whether participation is voluntary; iii) communicating the possibility of opting out or withdrawing from research at any given stage [56]. Thus, rolling informed consent results in a continuous consideration of the choices made by the vulnerable person [56].

## Conclusions

The principle of LLH like ALLEGRO is to connect technology developers with all users of the geriatric hospital environment, including patients with neurocognitive disorders. This implies using an efficient and pragmatic strategy to inform participants and obtain their consent, or their assent and the consent of their legally acceptable representative if applicable. The tool we offer here may be useful in the routine operation of LLH but can also be extended to all clinical trials in general. Further studies are needed to ensure the feasibility of this algorithm.

## Data Availability

There are no linked research data sets for this paper.

## Abbreviations

3D-CAM: 3-min Diagnostic interview for Confusion Assessment Method
4AT: 4 ‘A’s Test
ALLEGRO: Angers Living Lab En GéRiatrie hOpistalière
CAM: Confusion Assessment Method
GDPR: General Data Protection Regulation
LLH: Living Labs for Health
MacCAT-CR: MacArthur Competence Assessment Tool for Clinical Research
UBACC: University of California Brief Assessment of Capacity to Consent
